# The Effects of Antibiotics for *Helicobacter pylori* Eradication or Dapsone on Chronic Spontaneous Urticaria: A Systematic Review and Meta-Analysis

**DOI:** 10.3390/antibiotics10020156

**Published:** 2021-02-04

**Authors:** Jun Watanabe, Junya Shimamoto, Kazuhiko Kotani

**Affiliations:** Division of Community and Family Medicine, Jichi Medical University, 3311-1 Yakushiji, Shimotsuke-City, Tochigi 329-0498, Japan; m06105jw@jichi.ac.jp (J.W.); junyashimamoto@gmail.com (J.S.)

**Keywords:** antibiotics, chronic urticaria, dapsone, *Helicobacter pylori*, systematic review

## Abstract

Background: Chronic spontaneous urticaria (CSU) is a disease with wheals and/or angioedema. Some drugs, especially antibiotics for *Helicobacter pylori* (*H. pylori*) eradication and the sulfone antibiotic dapsone, may be candidates for treating CSU. The present study assessed the efficacy of these antibiotic therapies for CSU. Methods: Databases (MEDLINE, the Cochrane Central Register of Controlled Trials, EMBASE, the World Health Organization International Clinical Trials Platform Search Portal and ClinicalTrials.gov) were searched until October 2020. Study selection, data abstraction and quality assessments were independently performed using the Grading of Recommendations Assessment, Development and Evaluation approach. The outcomes were the remission of CSU-related symptoms, activities and adverse events due to antibiotics for *H. pylori* eradication or dapsone. Results: Nine randomized controlled trials (RCTs; 361 patients) were included. The antibiotics for *H. pylori* eradication increased the remission rate (risk ratio (RR) = 3.99, 95% confidence interval (CI) = 1.31 to 12.14; I^2^ = 0%), but dapsone did not (RR = 1.15, 95% CI = 0.74 to 1.78). Antibiotics for *H. pylori* eradication (standard mean difference (SMD) = 1.49, 95% CI = 0.80 to 2.18; I^2^ = 69%) and dapsone (SMD = 7.00, 95% CI = 6.92 to 7.08; I^2^ = 0%) improved symptoms. The evidence of certainty was moderate. Dapsone was associated with mild adverse events, whereas *H. pylori* eradication was not. Conclusion: Antibiotics, especially those for *H. pylori* eradication, improved the remission rate and symptoms of CSU with few adverse events. Further studies are needed.

## 1. Introduction

Chronic spontaneous urticaria (CSU) is a condition characterized by the development of wheals, angioedema or both and pruritus during a period of six or more weeks [1]. Chronic urticaria affects about 0.5–1% of the general population worldwide, and CSU accounts for over two-thirds of chronic urticaria cases, with affliction persisting for 20 years after the onset in up to 20% of patients [1,2,3]. CSU is not only an adult disease but also affects the pediatric population [4]. CSU thus has a significant impact on an impaired quality of life [5].

Previous systematic reviews showed that antibiotics for the eradication of chronic infections, such as *Helicobacter pylori* (*H. pylori*) infection, suppressed symptoms of CSU [6,7,8]. The antibiotics themselves might be more influential on this effect than the eradication of *H. pylori* [8]. The antibiotics used mainly for *H. pylori* eradication, such as amoxicillin, clarithromycin and metronidazole, may thus be closely involved in the pathology of CSU [7,8,9].

Dapsone is a sulfone antibiotic that can be used for antihistamine-refractory CSU; however, thus far, this drug is not fully recommended [1,2]. With this background, the quality of its supporting evidence in the guidelines was based on just one randomized control trial (RCT) and two case reports [10,11,12]. Therefore, the present study assessed the efficacy of antibiotics for chronic infections in CSU patients, especially antibiotics for *H. pylori* eradication and the sulfone antibiotic dapsone.

## 2. Methods

This review protocol was registered in protocol.io (https://www.dx.doi.org/10.17504/protocols.io.bq3qmymw, accessed on 4 February 2021), the Preferred Reporting Items for Systematic Review and Meta-Analyses (PRISMA) Statement [13].

### 2.1. Literature Search and Study Selection

Only RCTs were included in our assessment of the effects of oral antibiotics on CSU, irrespective of language, observation period, year of publication, publication and unpublished data. The inclusion criteria were patients with CSU, defined as recurrent attacks of hives daily or almost daily persisting for six or more weeks [1]. The patients over 16 years old were included as the dose of antibiotics used at age 16 is generally the same as that for adults [14]. Oral antibiotics included the *H. pylori* eradication regimen and the sulfone antibiotic dapsone with immunomodulatory agents. The comparators were the corresponding placebo or no placebo. The outcomes were remission rates, improvement of CSU symptoms and adverse events. Remission of urticaria symptoms was defined as no attacks of wheals or pruritus at the end of the follow-up period [1]. The improvement of urticaria symptoms was defined by the difference in the urticaria activity score (UAS), which is the sum of the hives score and itch severity score, between the baselines and the end of follow-up [1]. When the total score was unknown, the urticaria (hives) score was adopted.

The electronic databases were searched using the Cochrane Central Register of Controlled Trials (CENTRAL), MEDLINE via Ovid and EMBASE via PROQUEST (Appendix B). The clinical trial registrations were searched using the website of the World Health Organization International Clinical Trials Platform Search Portal (ICTRP) and ClinicalTrials.gov (Appendix B). The reference lists of guidelines were searched for articles related to urticaria [1,2,3]. The reference lists were also hand-searched from the included trials. The authors were contacted to obtain further data as necessary.

### 2.2. Data Extraction and Quality Assessments

Two review authors (J.W. and J.S.) independently completed the construction of databases, article screening, study quality assessment and data extraction. Discrepancies were resolved by discussion with the third review author (K.K.). Data were extracted using a standardized data extraction form. Information collected included the characteristics (author, year, country, patient number, age, antibiotics, control, follow-up periods and eradication rate) and data (outcomes) from the included studies. The risk of bias was assessed using version 2 of the Cochrane risk-of-bias tool for randomized trials (RoB2) tool [15].

### 2.3. Data Synthesis and Analyses

Relative risks with 95% confidence intervals (CIs) were calculated for the remission rate. The standard mean differences (SMD) and 95% CIs were calculated for the improvement of CSU symptoms. All adverse events were summarized according to the definition of each study.

The statistical heterogeneity was first assessed by a visual inspection of the forest plot and using the I^2^ statistic (I^2^ values of 0% to 40%: may not be important; 30% to 60%: may represent moderate heterogeneity; 50% to 90%: may represent substantial heterogeneity; 75% to 100%; considerable heterogeneity) [15]. When heterogeneity was identified (I^2^ statistic > 50%), possible sources of heterogeneity were explored using subgroup analyses. The publication bias was evaluated by searching the clinical trial registries, but the funnel plot asymmetry was not assessed because the number of studies in each meta-analysis was <10 according to the Cochrane handbook [15]. In studies of *H. pylori* eradication, the subgroup analyses were *H. pylori* eradication rate: ≥80% versus <80% [6]. The following sensitivity analyses were performed: (1) only the participants who completed the study with full data and (2) exclusion of studies with a high overall risk of bias.

Meta-analyses with a random-effects model were performed using the Review Manager software program (RevMan 5.4.1). The findings for the outcomes were summarized in a table based on the Cochrane handbook [15]. The quality of evidence was evaluated by the Grading of Recommendations Assessment, Development and Evaluation (GRADE) approach [16].

## 3. Results

### 3.1. Study Selection

**Figure 1:**
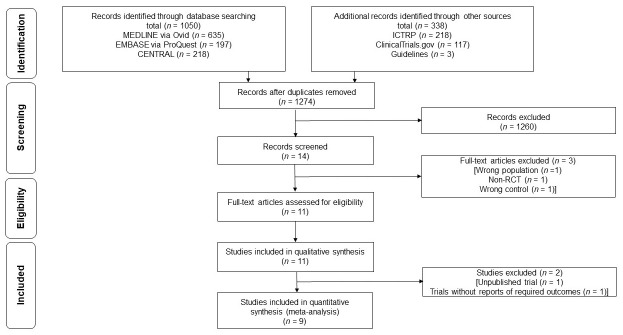
Flow diagram of the literature search results.

Figure 1 shows the selection process. After the initial screening of titles and abstracts with 1274 records until 4 October 2020, 14 records were identified. After the full-text screening, three studies were excluded because of participants having urticaria but not chronic urticaria [17], non-RCTs [18] and inappropriate controls [19]. A total of 10 RCTs (including one trial without publication (IRCT2016082114333N56 [20])) were detected for the qualitative synthesis. Ultimately, 9 trials with 361 participants were included in the quantitative synthesis because 1 trial did not report the remission rate or clinical improvement of urticarial-related symptoms in the control groups [21].

Table 1 summarizes the characteristics of the studies included in the quantitative synthesis [10,22,23,24,25,26,27,28,29]. Antibiotics were used in the *H. pylori* eradication regimen in six trials, while dapsones was used in three trials. The follow-up periods were within three months in five trials and beyond three months in four trials. Table 2, Table A1 and Table A2 show the risk of bias. Overall, only one study had a high risk of bias due to deviations from intended interventions, missing outcome data and an unclear measurement of the outcomes.

### 3.2. Outcomes

Table 3 shows the summary of findings, which include an overall grading of the evidence related to each of the outcomes using the GRADE approach [16].

#### 3.2.1. Remission Rate

**Figure 2:**
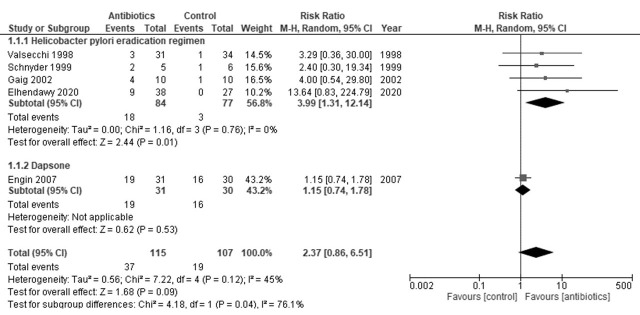
Forest plot of the remission rate.

Regarding the remission rate of CSU, four studies on *H. pylori* and one study on dapsone were identified [10,22,23,24,27]. The *H. pylori* eradication regimen increased the remission rate of CSU (RR = 3.99, 95% CI = 1.31 to 12.14; I^2^ = 0%) (Figure 2) [22,23,24,27]. Dapsone resulted in little to no difference in the remission rate (RR = 1.15, 95% CI = 0.74 to 1.78) [8]. The evidence of certainty was moderate.

#### 3.2.2. Clinical Improvement

**Figure 3:**
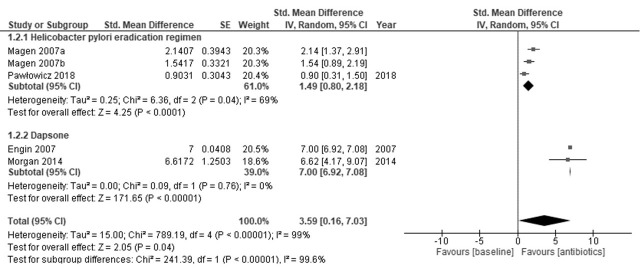
Forest plot of the clinical improvement in antibiotics.

**Figure 4:**
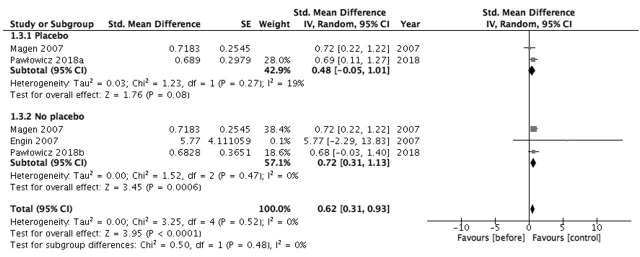
Forest plot of the clinical improvement in controls.

Regarding the improvement in CSU, two studies on *H. pylori* and two studies on dapsone were identified [10,25,26,29]. The *H. pylori* eradication regimen resulted in a slight increase in clinical improvement (SMD = 1.49, 95% CI = 0.80 to 2.18; I^2^ = 69%) (Figure 3) [22,23]. Dapsone resulted in a large increase in clinical improvement (SMD = 7.00, 95% CI = 6.92 to 7.08; I^2^ = 0%) [26,29]. Controls showed little to no effect on the clinical improvement of CSU (SMD = 0.62, 95% CI = 0.31 to 0.93; I^2^ = 0%) (Figure 4) [10,25,26,29]. The evidence of certainty was moderate.

#### 3.2.3. Adverse Events

One study on *H. pylori* and three studies on dapsone were identified. One study on *H. pylori* and one study on dapsone reported no adverse events [10,13,28,29]. One study on dapsone reported that 3/38 (7.9%) patients in dapsone groups had drug-related symptoms (nausea in 2 patients and fatigue and headache in 1 patient), and 1/27 (3.7%) patients in control groups had gastrointestinal upset [10]. In another study on dapsone, 4/10 (40%) patients in dapsone groups had adverse events (nausea in 2 patients, vaginal candidiasis in 1 patient and mild neuropathy in 1 patient), and 3/12 (25%) patients in control groups had viral respiratory infections [29]. The evidence of certainty was low.

### 3.3. Additional Analyses

**Figure A1:**
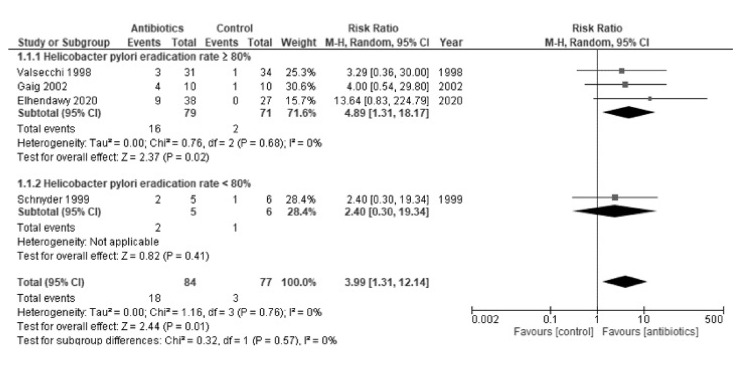
Forest plot of the remission rate with *H. pylori* eradication rate: ≥80% versus <80%.

**Figure A2:**
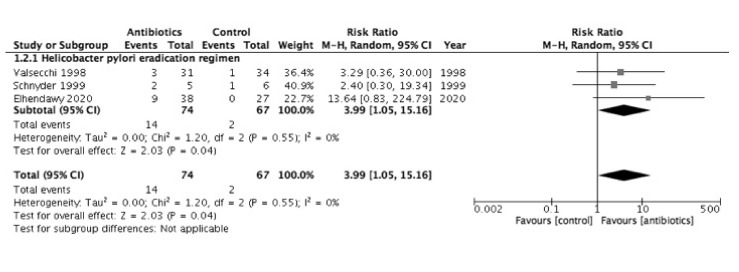
Forest plot of the remission rate including only studies of the participants who completed the study with full data.

**Figure A3:**
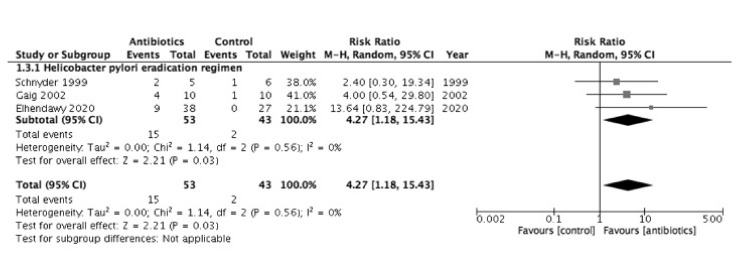
Forest plot of the remission rate excluding studies with a high overall risk of bias.

In the subgroup analysis of the *H. pylori* eradication rate, an *H. pylori* eradication rate ≥80% (RR = 4.89, 95% CI = 1.31 to 18.17; I^2^ = 0%) increased the remission rate of CSU, but not an *H. pylori* eradication rate <80% (RR = 2.40, 95% = CI 0.30 to 19.34) (Figure A1). The prespecified sensitivity analyses were consistent with the primary findings (Figure A2 and Figure A3).

## 4. Discussion

*H. pylori* infection is thought to have a potential trigger for CUS, and it is thus searched during the diagnostic work-up of CSU, even though the epidemiological/clinical evidence is not very strong, or at least inconsistent [30,31]. In the present systematic review and meta-analyses, antibiotic therapy for *H. pylori* eradication increased the remission rate and induced improvement in CSU with few adverse events. Dapsone might not increase the remission rates and could improve CSU with mild adverse events. Given the results of RCTs only, both antibiotics were recognized to be useful for CSU.

There is an earlier review showing that antibiotics for *H. pylori* eradication suppressed CSU symptoms and improved remission with or without *H. pylori* eradication [8]. In our subgroup analysis, high eradication rates of *H. pylori* increased remission of CSU, while low eradication rates did not. Keeping such observations in mind, we consider several hypotheses concerning the mechanisms of the effect of antibiotics on CSU: (1) alleviation of inflammation, (2) favorable modulation of gut microbiota or (3) eradication of *H. pylori* itself (in CUS patients with *H. pylori* infection) [32]. First, CUS-related systemic inflammation is caused by activated mast cells and induced cytokines [33,34]. Some antibiotics, such as clarithromycin, attenuate proinflammatory cytokine production during the innate immune response by inhibiting Th2 cytokine secretion [35]. Second, the diversity of the microbiota is a regulator in the gut–skin axis [36,37,38,39]. Antibiotics can modulate favorably the microbiota, resulting in the reduction of systemic inflammation [40]. In addition, as *H. pylori* itself induced the release of histamine by mast cells, *H. pylori* eradication by antibiotics would have a favorable effect on the pathophysiology of CSU [41,42].

In the guidelines that included only one RCT in Europe, dapsone had little evidence on CSU [1,2]. The present review included three RCTs, suggesting that dapsone might not increase the remission rates but did improve the symptoms. The significant improvement in the symptoms by dapsone is important because patients with CSU have an impaired quality of life due to their symptoms [43]. The mechanism underlying the effects of dapsone on CSU remains unclear, but dapsone cannot affect *H. pylori*. The various anti-inflammatory effects of dapsone seem to be involved; namely, dapsone prevents the production of 5-lipoxygenase products in neutrophils, downregulates leukotrienes and inhibits prostaglandin and leukotriene activities [44,45,46,47,48].

Our present finding that adverse events may be more likely to occur with dapsone than with *H. pylori* eradication should be interpreted with caution. In our review, only one of the six studies concerning *H. pylori* eradication reported adverse events, while all three studies concerning dapsone reported these data. In previous systematic reviews, antibiotic therapy for *H. pylori* eradication increased the rate of mild adverse events, such as gastrointestinal symptoms, compared to no eradication therapy [49,50]. Furthermore, dapsone has been safely used in the long term as a treatment for leprosy. However, patients using dapsone require regular examinations, as this agent causes some rare but major adverse events, such as met-hemoglobinemia, hemolysis, agranulocytosis and peripheral neuropathy with primarily motor function loss [43].

The present review has strengths compared to the previous systematic reviews [6,7,8]. First, a rigorous methodology was adopted according to the PRISMA statement [13], including a comprehensive search and duplicate assessments for evidence. Second, the GRADE approach was used to assess the certainty of the evidence [16]. Although the certainty was very low in a previous systematic review [6], the present review showed moderate evidence and provided a practical estimate of the effects of antibiotics for CUS. The present review had several limitations. First, this review included only antibiotics for *H. pylori* eradication and dapsone. Although previous studies have largely focused on antibiotics of *H. pylori* itself and inflammation, further studies focusing on three mechanisms, including gut microbiota, are needed. As candidates of other antibiotics, tetracycline used in bismuth quadruple therapy has a high eradication rate of *H. pylori* and improves urticaria [17,51]. Second, all studies included in this review had a small sample size. Further large-scale studies are needed to increase the certainty and generalizability of the evidence.

## 5. Conclusions

In conclusion, the present systematic review and meta-analysis showed that antibiotic therapies, especially those for *H. pylori* eradication, improved the remission rates and symptoms of CSU with few adverse events. These findings provide relevant information that physicians can use antibiotics for CSU. Further studies are warranted to assess the efficacy and safety of antibiotics on CSU.

## Figures and Tables

**Table 1:** Summary of the characteristics of the eligible studies.

Authors (Ref No.)	Year	Country	Subject No.	Age (Years)	Antibiotics (Months)	Control	Follow-Up (Months)	Eradication Rate (%)
Valsecchi [22]	1998	Italy	65	24–61	CMO (7)	No placebo	12	93.5
Schnyder [23]	1999	Switzerland	11	34–73	AL (14)	Placebo	2	20.0
Gaig [24]	2002	Spain	16	27–55	ACO (7)	Placebo	1.5	100
Magen [25]	2007	Israel	78	42.5 (6.8) *	ACO (14)	No placebo	4	86.7
Pawłowicz [26]	2018	Poland	64	42.8 (13.3) **	ACO (7)	Placebo/no placebo	6	91.7
El-hendawy [27]	2020	Egypt	27	30.9 (7.9)	ACO (14)	Placebo	2	85.7
Engin [10]	2007	Turkey	65	16–60	Dapsone	No Placebo	6	-
Rajan [28]	2010	United Kingdom	13	-	Dapsone	Placebo	4	-
Morgan [29]	2014	USA	22	25–64	Dapsone	Placebo	1.5	-

A, amoxicillin; C, clarithromycin; L, lansoprazole; M, metronidazole; O, omeprazole. * Age of the group with positive autologous serum skin test and ^13^C-urea breath test findings. ** Age of all participants enrolled in the study.

**Table 2:** Quality scores for the eligible studies for remission rate.

Authors (Ref No.)	Risk of Bias 2 Tool Assessment					
	Bias Arising from the Randomization Process	Bias due to Deviations from Intended Interventions	Bias due to Missing Outcome Data	Bias in Measurement of the Outcome	Bias in Selection of the Reported Results	Overall Risk of Bias
Valsecchi [22]	Some concerns	High	High	High	Some concerns	High
Schnyder [23]	Some concerns	Low	Low	Low	Some concerns	Some concerns
Gaig [24]	Some concerns	Low	Low	Low	Some concerns	Some concerns
Elhendawy [27]	Low	Low	Low	Low	Some concerns	Some concerns
Engin [10]	Low	Some concerns	Low	Some concerns	Some concerns	Some concerns

**Table 3:** Summary of findings.

The Efficacy and Safety of Antibiotics for Chronic Spontaneous Urticaria						
Patient or Population: Adults Setting: Chronic Spontaneous Urticaria Intervention: Antibiotics Comparison: Control						
Outcomes	Anticipated Absolute Effects * (95% CI)		Relative Effect(95% CI)	Patient Number(Studies)	Certainty of the Evidence (GRADE)	Comments
	Risk with Control	Risk with Antibiotics				
Remission rate	178 per 1000	421 per 1000 (153 to 1000)	RR = 2.37 (0.86 to 6.51)	222 (5 RCTs)	Moderate ^a^	The *H. pylori* eradication regimen likely increased the remission rate of chronic urticaria, but not dapsone.
Clinical improvement	-	SMD 3.59 SD higher (0.16 to 7.03)	-	229 (4 RCTs)	Moderate ^a^	Antibiotics (*H. pylori* eradication regimen and dapsone) likely increased the improvement of chronic urticaria.
Adverse events	0% in a study of *H. pylori* eradication, 11.5% in 3 studies of dapsone.			117 (5 RCTs)	Low ^a,b^	Dapsone had mild adverse events, such as nausea, fatigue, and headache.

CI, confidence interval; RR, risk ratio; SMD, standard mean difference. * The risk in the intervention group (and its 95% CI) is based on the assumed risk in the comparison group and the relative effect of the intervention (and its 95% CI). GRADE Working Group grades of evidence: High certainty: We are very confident that the true effect lies close to that of the estimated effect. Moderate certainty: We are moderately confident in the estimated effect. The true effect is likely to be close to the estimated effect, but there is a possibility that it is substantially different. Low certainty: Our confidence in the estimated effect is limited. The true effect may be substantially different from the estimated effect. Very low certainty: We have very little confidence in the estimated effect. The true effect is likely to be substantially different from the estimated effect. ^a^ Downgraded because of imprecision due to the small sample size. ^b^ Downgraded because of a high risk of bias because the assessment was likely influenced by knowledge of intervention.

## Abbreviations

CI = confidence interval, CSU = chronic spontaneous urticaria, *H. pylori* = *Helicobacter pylori*, RCT = randomized controlled trials, RR = risk ratio, SMD = standard mean difference.

## Appendix A

**Table A1:** Quality scores for the eligibility studies for clinical improvement.

Authors. (Ref No.)	Risk of Bias 2 Tool Assessment					
	Bias Arising from the Randomization Process	Bias due to Deviations from Intended Interventions	Bias due to Missing Outcome Data	Bias in Measurement of the Outcome	Bias in Selection of the Reported Results	Overall Risk of Bias
Magen [25]	Some concerns	Low	Low	Some concerns	Some concerns	Some concerns
Pawłowicz [26]	Some concerns	Low	Low	Low	Some concerns	Some concerns
Engin [10]	Some concerns	Low	Low	Some concerns	Some concerns	Some concerns
Morgan [29]	Low	Low	Low	Low	Some concerns	Some concerns

**Table A2:** Quality scores for the eligibility studies for adverse events.

Authors (Ref No.)	Risk of Bias 2 Tool Assessment					
	Bias Arising from the Randomization Process	Bias due to Deviations from Intended Interventions	Bias due to Missing Outcome Data	Bias in Measurement of the Outcome	Bias in Selection of the Reported Results	Overall Risk of Bias
Gaig [24]	Some concerns	Low	Low	Low	Some concerns	Some concerns
Engin [10]	Some concerns	Low	Low	High	Some concerns	High
Rajan [28]	Some concerns	Low	High	High	Some concerns	High
Morgan [29]	Low	Low	Low	Low	Some concerns	Some concerns

## Appendix B

Search strategy of the electronic databases

***CENTRAL search strategy***

1. MeSH descriptor: [Urticaria] explode all trees
2. urticaria:ti,ab,kw (Word variations have been searched)
3. hives:ti,ab,kw (Word variations have been searched)
4. 1. OR 2. OR 3.
5. MeSH descriptor:[Anti-Bacterial Agents]explode all tree
6. 6 (antibiotic* or antibacteri* or anti* bacter* or bacteriocid* or bactericid* or anti*microbial or ciprofloxacin or metronidazole or levamisole or ornidazole or fusidin or rifaximin or vancomycin or fusidic acid or nitazoxanide or teicoplanin or rifampicin or bacitracin or fidaxomicin or amoxicillin or azithromycin or cephalosporin* or cephalexin or clarithromycin or clindamycin or doxycycline or erythromycin or fluoroquinolone* or levofloxacin or macrolide* or nitrofurantoin or penicillin or tetracycline or trimethoprim or dapsone):ti,ab,kw (Word variations have been searched)
7. 5. OR 6
8. MeSH descriptor: [Helicobacter] explode all trees
9. MeSH descriptor: [Helicobacter Infections] explode all trees
10. MeSH descriptor: [Helicobacter pylori] explode all trees
11. 11 (helicobacter or pylori or pyloridis or Campylobacter):ti,ab,kw (Word variations have been searched)
12. 8. OR 9. OR 10. OR 11.
13. 13 7. OR 12.
14. 4. AND 13.

***MEDLINE (via Ovid) search strategy***

1. exp Urticaria/
2. urticaria.tw.
3. hives.tw.
4. or/1–3
5. exp Anti-bacterial Agents/
6. (antibiotic$ or antibacteri$ or anti$bacter$ or bacteriocid$ or bactericid$ or anti$microbial or ciprofloxacin or metronidazole or levamisole or ornidazole or fusidin or rifaximin or vancomycin or fusidic acid or nitazoxanide or teicoplanin or rifampicin or bacitracin or fidaxomicin or amoxicillin or azithromycin or cephalosporin$ or cephalexin or clarithromycin or clindamycin or doxycycline or erythromycin or fluoroquinolone$ or levofloxacin or macrolide$ or nitrofurantoin or penicillin or tetracycline or trimethoprim or dapsone).tw.
7. or/5–6
8. exp Helicobacter pylori/ or exp Helicobacter/ or exp Helicobacter infection/
9. (pylori or pyloridis or HP).tw.
10. (helicobacter or Campylobacter).tw.
11. or/8–10
12. 7 or 11
13. 4 and 12
14. ((randomized controlled trial or controlled clinical trial).pt. or randomized.ab. or randomised.ab. or placebo.ab. or drug therapy.fs. or randomly.ab. or trial.ab. or groups.ab.) not (exp animals/ not humans.sh.)
15. 13 and 14

***EMBASE (via PROQUEST) search strategy***

- S1. EMB.EXACT.EXPLODE(“chronic urticaria”)
- S2. (ab(urticaria) OR ti(urticaria))
- S3. (ab(hives) OR ti(hives))
- S4. S1 OR S2 OR S3
- S5. EMB.EXACT.EXPLODE(“antibiotic agent”)
- S6. (ab(antibiotic) OR ti(antibiotic) OR ab(antibiotics) OR ti(antibiotics))
- S7. (ab(antibacterial) OR ti(antibacterial) OR ab(anti bacteral) OR ti(anti bacteral))
- S8. (ab(bacteriocidal) OR ti(bacteriocidal) OR ab(bactericidal) OR ti(bactericidal) OR ab(anti microbial) OR ti(anti microbial))
- S9. (ab(ciprofloxacin) OR ti(ciprofloxacin) OR ab(metronidazole) OR ti(metronidazole) OR ab(levamisole) OR ti(levamisole) OR ab(ornidazole) OR ti(ornidazole) OR ab(fusidin) OR ti(fusidin) OR ab(rifaximin) OR ti(rifaximin) OR ab(vancomycin) OR ti(vancomycin) OR ab(fusidic acid) OR ti(fusidic acid) OR ab(nitazoxanide) OR ti(nitazoxanide) OR ab(teicoplanin) OR ti(teicoplanin) OR ab(rifampicin) OR ti(rifampicin) OR ab(bacitracin) OR ti(bacitracin) OR ab(fidaxomicin) OR ti(fidaxomicin) OR ab(amoxicillin) OR ti(amoxicillin) OR ab(azithromycin) OR ti(azithromycin) OR ab(cephalosporin) OR ti(cephalosporin) OR ab(cephalosporinic) OR ti(cephalosporinic) OR ab(cephalexin) OR ti(cephalexin) OR ab(clarithromycin) OR ti(clarithromycin) OR ab(clindamycin) OR ti(clindamycin) OR ab(doxycycline) OR ti(doxycycline) OR ab(erythromycin) OR ti(erythromycin) OR ab(flouroquinolone) OR ti(flouroquinolone) OR ab(levofloxacin) OR ti(levofloxacin) OR ab(macrolide) OR ti(macrolide) OR ab(nitrofurantoin) OR ti(nitrofurantoin) OR ab(penicillin) OR ti(penicillin) OR ab(tetracycline) OR ti(tetracycline) OR ab(trimethoprim) OR ti(trimethoprim) OR ab(dapsone) OR ti(dapsone))
- S10. S5 OR S6 OR S7 OR S8 OR S9
- S11. EMB.EXACT.EXPLODE(“Helicobacter pylori”)
- S12. EMB.EXACT.EXPLODE(“Helicobacter”)
- S13. EMB.EXACT.EXPLODE(“Helicobacter infection”)
- S14. (ab(pylori or pyloridis or HP) OR ti(pylori or pyloridis or HP))
- S15. (ab(helicobacter or Campylobacter) OR ti(helicobacter or Campylobacter))
- S16. S11 OR S12 OR S13 OR S14 OR S15
- S17. S10 OR S16
- S18. S4 AND S17
- S19. (ab(random*) OR ti(random*)) OR (ab(placebo*) OR ti(placebo*)) OR (ab(double NEAR/1 blind*) OR ti(double NEAR/1 blind*))
- S20. S18 AND S19

***ClinicalTrials.gov. search strategy***

Condition or disease: chronic urticaria

***ICTRP search strategy***

chronic urticaria
